# Role of ubiquitin-specific proteases in programmed cell death of breast cancer cells

**DOI:** 10.1016/j.gendis.2024.101341

**Published:** 2024-06-03

**Authors:** Wen Yan, Shasha Xiang, Jianbo Feng, Xuyu Zu

**Affiliations:** The First Affiliated Hospital, Cancer Research Institute, Hengyang Medical School, University of South China, Hengyang, 421001 Hunan, China

**Keywords:** Anoikis, Apoptosis, Autophagy, Breast cancer, Ferroptosis, Pyroptosis, Ubiquitin-specific proteases

## Abstract

Breast cancer (BC) is the most common malignant tumor and the leading cause of cancer-related deaths among women worldwide. Great progress has been recently achieved in controlling breast cancer; however, mortality from breast cancer remains a substantial challenge, and new treatment mechanisms are being actively sought. Programmed cell death (PCD) is associated with the progression and treatment of many types of human cancers. PCD can be divided into multiple pathways including autophagy, apoptosis, mitotic catastrophe, necroptosis, ferroptosis, pyroptosis, and anoikis. Ubiquitination is a post-translational modification process in which ubiquitin, a 76-amino acid protein, is coupled to the lysine residues of other proteins. Ubiquitination is involved in many physiological events and promotes cancer development and progression. This review elaborates the role of ubiquitin-specific protease (USP) in programmed cell death, which is common in breast cancer cells, and lays the foundation for tumor diagnosis and targeted therapy.

## Introduction

According to the latest data from the International Agency for Research on Cancer (IARC) of the World Health Organization, breast cancer (BC) is one of the most prevalent malignancies worldwide. It is the primary cancer that affects women^1^ and is the leading cause of cancer-related deaths in women aged 20–39 years.^2^ Recently, various types of BC, as well as the key molecular drivers and prognostic features of BC, have been identified.^3^ Despite recent substantial advances in controlling BC, the rate of decline in female BC mortality is gradually slowing down.^2^ To optimize the multi-selectivity of therapies and discover more mechanisms to guide therapeutic decisions, this review provides a summary in the context of deubiquitination and programmed cell death (PCD), which are crucial processes in cancer development and progression.

Ubiquitination is a post-translational modification process by which ubiquitin, a 76-amino acid protein, is linked to the lysine residues of other proteins. Ubiquitination is not only involved in a wide range of physiological events but also promotes the development and progression of cancer.^4^ Deubiquitination is the reverse process of ubiquitination and requires the removal of UB from the substrate, catalyzed by a class of deubiquitinases (DUBs), with ubiquitination and deubiquitination constituting a dynamic equilibrium in cell biology. To date, more than 100 DUBs have been identified,^5^ and by removing UB from the substrate, DUB can rescue specific proteins from degradation markers and maintain their protein stability.^4^

The ubiquitin-specific protease (USP) is the largest family of DUB.^6^ It recognizes the ubiquitination signals of specific proteins, leading to the deubiquitination of target proteins. These proteins are involved in various biological functions, such as cell proliferation, differentiation, apoptosis, and migration. Aberrant expression or activity of certain USPs is strongly linked to the development and progression of human tumors. Therefore, some USPs have been utilized as novel molecular tumor markers and therapeutic targets.

Programmed cell death, also known as regulated cell death (RCD),^7^ is a conserved evolutionary process of cellular suicide that is critical for the development and integrity of organisms. Dysregulation of this program has been linked to a variety of diseases, including cancer.^8^ An interconnection has been recently revealed between the ubiquitin-proteasome system (UPS) and the programmed cell death system, although they operate independently.

This review summarizes the main classifications of PCD and its role in BC. It then outlines the functional mechanisms of USPs and PCD in BC, which may aid readers in understanding the impact of PCD and USPs on BC development, as well as the role of USPs in BC development by affecting PCDs. This review may also help identify new therapeutic targets for BC.

## Role of PCD in BC

**Figure 1:**
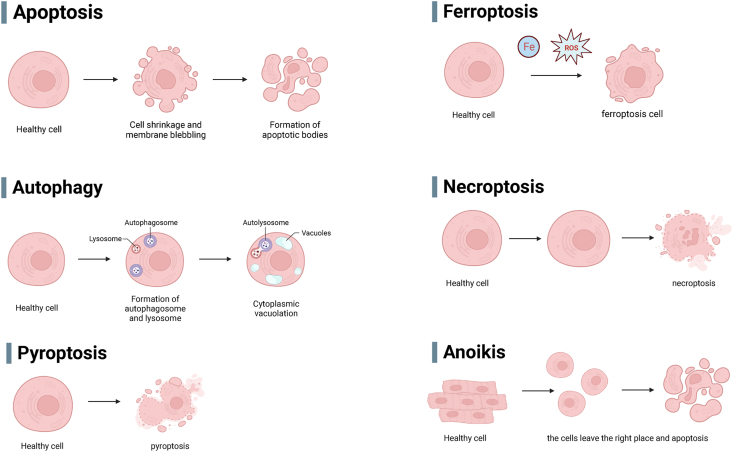
Different types of programmed cell death. This picture summarizes the different pathways of programmed cell death (created with BioRender.com) (Note: ROS: reactive oxygen species).

PCD is considered a strict form of RCD,^9^ and this form of RCD is orchestrated by a number of evolutionarily conserved pathways that have important implications for developmental processes and immune responses.^10^ PCD identified in current studies includes apoptosis, autophagy, pyroptosis, ferroptosis, mitotic catastrophe, necroptosis, and anoikis (Fig. 1).^7^^,^^11^ In cancer, malignant cells produce an excess of reactive oxygen species (ROS),^12^ Therapeutic strategies that utilize oxidative stress can kill cancer cells by triggering PCD, the failure of which may lead to uncontrolled cell proliferation and play a key role in cancer.^13^ PCD is a natural barrier to carcinogenesis, whereas apoptosis is attenuated in high-grade malignant tumors and treatment-resistant state in several studies.^14^^,^^15^ Aberrant molecular mechanisms of apoptotic signaling trigger BC cells to re-enter the apoptotic cycle. This may be a key pathway for the treatment of BC. Dysregulation of autophagy has been implicated in BC pathogenesis and metastasis.^16^^,^^17^ Different subtypes of BC have different susceptibility to ferroptosis, and induction of ferroptosis may effectively overcome therapeutic resistance in BC.^18^ Pyroptosis modifies the tumor microenvironment in BC and impacts BC progression and therapeutic strategies.^19^ Anoikis has been mainly studied in BC metastasis, especially in triple-negative breast cancer. A deeper study of PCD in BC shows that PCD plays a multifunctional role in biological processes in BC. Next, we describe the roles of the different types of PCD in BC.

## Apoptosis in BC

The breast undergoes two stages of morphological development: puberty and pregnancy stages, during which the proliferation and differentiation of mammary cells are substantially altered and are affected by the Bcl-2 family that regulates apoptosis.^20^ In normal breast cells, a balance is present between proliferation and apoptosis, anti-apoptosis and pro-apoptosis,^21^ which maintains cellular homeostasis; once they are out of balance, activation of the anti-apoptotic pathway or defects in the pro-apoptotic path can lead to uncontrolled cell proliferation, therapeutic resistance, and recurrence of the cancer cells.^22^ Many mechanisms can induce apoptosis in BC cells, such as the mitochondrial pathway, PIK3K/AKT, NFκB.^23^ Apoptosis is identified as a regulatory process that promotes BC cell death.

## Autophagy in BC

Autophagy can be divided into four categories: macroautophagy, microautophagy, chaperone-mediated autophagy, and selective autophagy.^24^ In normal breast cells and tissues, autophagy plays an essential role in the development and differentiation of luminal structures and maintenance of homeostasis in vivo.^25^ The expression of autophagy-related genes is higher in normal mammary glands than that in BC cells.^26^ In this regard, autophagy is thought to be suppressive, e.g. autophagy inhibits the progression of HER2-mediated BC.^27^ However, when the tumor reaches an advanced stage, autophagy promotes tumor progression and makes it resistant to treatment.^28^ Here, autophagy demonstrates a promoting role in cancer. Therefore, autophagy is a “double-edged sword” in BC.

## Ferroptosis in BC

Ferroptosis is a recently discovered form of non-apoptotic PCD, which is mainly caused by iron overload and reactive oxygen species-dependent accumulation of lipid peroxides.^7^ Ferroptosis occurs in BC, and ferroptosis inducers increase BC cell death.^29^ Reports of ferroptosis have focused on triple-negative breast cancers, which are most prone to recurrence and drug resistance,^30^, ^31^, ^32^ and some researchers have recently found that triple-negative breast cancers are more sensitive to ferroptosis than ER-positive breast cancers.^33^ BC cell death can be synergistically induced by disrupting cellular iron metabolism and redox homeostasis.^34^^,^^35^ Moreover, ferroptosis sensitivity can be determined by building lipid composition.^33^^,^^36^ The role of p53 in ferroptosis has been also well-established.^37^, ^38^, ^39^

## Pyroptosis in BC

Pyroptosis is an inflammatory PCD mediated by an inflammasome that cleaves gasdermin family proteins and activates cytokines, such as IL-1β.^40^ As an inflammatory cell death mechanism, vital elements in pyroptosis, such as the inflammasome, gasdermin proteins, and inflammatory cytokines, are involved in malignant neoplastic transformation and development. Inflammatory cytokines, such as IL-1β, released after activation of pyroptosis can promote the development of a variety of malignant tumors, including BC.^41^ Pyroptosis has been shown to inhibit tumor growth in non-small-cell lung cancer (NSCLC) and osteosarcoma.^42^^,^^43^ Based on the available studies, it can be concluded that cellular pyroptosis generates a microenvironment that promotes the processes of tumor formation and progression, including tumor growth, invasion, and metastasis. In contrast, the induction of cellular pyroptosis can inhibit cancer development and progression.

## Necroptosis and anoikis in BC

Necroptosis is a novel form of PCD that is mainly mediated by receptor-interacting serine/threonine kinase protein (RIPK) 1, RIPK3, and MLKL (Mixed lineage kinase domain-like protein).^44^ Necroptosis not only prevents tumor development but also promotes tumor progression by triggering an inflammatory response.^45^, ^46^, ^47^ Models have been developed based on seven necroptosis-associated LncRNAs to predict BC prognosis^48^ or metastasis based on necroptosis-associated miRNAs.^49^ Necroptosis has not been well-studied in BC, and the regulation of tumor necroptosis may be a modality with potential therapeutic strategies, and more studies are expected in the future.

The disruption of normal epithelial cell-extracellular matrix interactions leading to apoptosis is termed anoikis.^50^ Cadherins, the proteins responsible for cell-cell and cell-epithelial adhesion, are expressed in the breast,^51^^,^^52^ suggesting that cadherins induce anoikis and that anoikis is essential to inhibit cellular colonization and growth into the new stromal environment.^53^ When induced, anoikis may positively regulate BC development, particularly tumor metastasis.

Various types of PCDs have been found to have specific roles in different aspects of BC development, metastasis, and drug resistance (Table 1). The current challenge is to inhibit the proliferation, metastasis, and drug resistance of BC cells by regulating PCD. Some types of PCD play a dual role in BC development. Additionally, the crosstalk between various types of PCD, such as ferroptosis and autophagy, is noteworthy. The precise targeting and regulation of PCD and its modification for the treatment of BC requires further in-depth research.

**Table 1:** PCD in BC.

Type of PCD	The main function aspect	Reference
Apoptosis	proliferation	20
	differentiation	
	therapeutic resistance	22
Autophagy	differentiation	25
	development of luminal structures	
	therapeutic resistance	28
Ferroptosis	therapeutic resistance	30, 31, 32, 33
Pyroptosis	malignant neoplastictransformation	41, 42, 43
	invasion	
	metastasis	
	proliferation	
Necroptosis	predict prognosis	48
	metastasis	49
Anoikis	metastasis	53

(Note: PCD: programmed cell death; BC: breast cancer).

## USPs and PCD in BC

### The basic structure and functional mechanism of USPs

**Figure 2:**
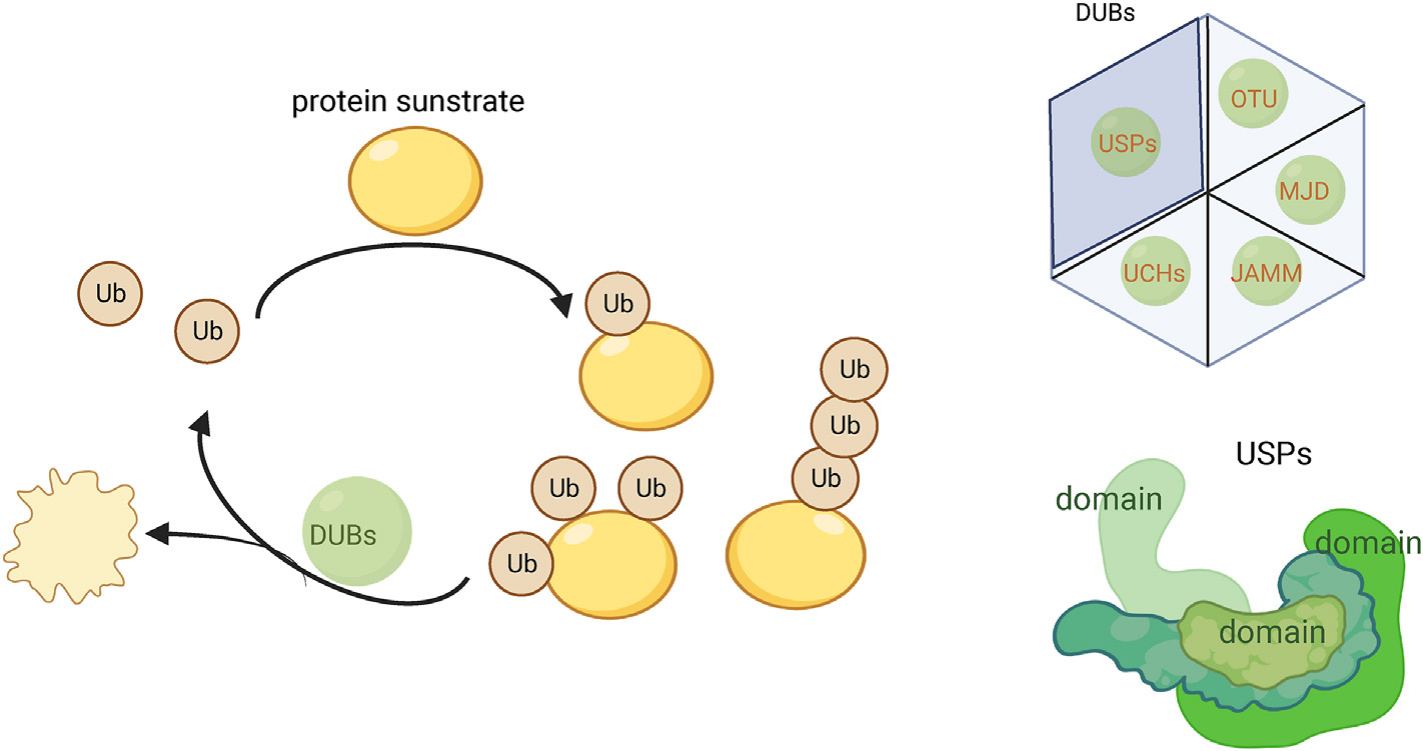
Overview of the process of ubiquitination and the structure of ubiquitin specific protease. This figure shows the process of deubiquitination and the classification enzymes, as well as the simplified structure of USP (created with BioRender.com). (Note: Ub: ubiquitin; DUBs: deubiquitinases; UCHs: ubiquitin C-terminal hydrolases; OUT: ovarian tumor proteases; MJD: Machado-Joseph domain proteases; JAB1/MPN/MOV34 metalloproteases; USP: ubiquitin specific protease).

USPs represent the majority of DUBs encoded by the human genome and are the most prominent family of DUBs,^6^ a class of cysteine-dependent proteins with a mechanism of action similar to that of the cysteine protease papain. They all have highly conserved USP structural domains ranging in size from 300 to 800 amino acids.^54^ They all have highly conserved USP domains formed by three subdomains resembling the palm, thumb, and fingers of a right hand. The catalytic site is located between the palm and thumb structural domains, and the finger structural domains are responsible for interacting with distal ubiquitin.^55^ In addition to the above three structural domains, it has been proposed that the structural domains of USP can also be defined as the following: ubiquitin-associated domain (UBA), ubiquitin-interacting motif (UIM), and zinc finger ubiquitin-specific protease domain (ZnF-UBP), USP-specific structural domain (DUSP), and ubiquitin-like structural domain (UBL) (Fig. 2). The catalytic core is in the DUSP.^56^ These structural domains confer specificity for the USP binding to substrates. These domains may regulate enzymatic activity and interact with proteins.

There is a relative structural diversity among USPs, but the catalytic domains are highly conserved, and there is no major difference in the catalytic ability of USPs. Thus, the catalytic ability of USPs mainly depends on the nucleophilic attack of cysteines at the catalytic site.^57^ USPs exert deubiquitination by binding the proteins asscociated with cell cycle progression, modulating c-Myc, stabilizing, regulating apoptosis-associated factors, and participating in DNA damage repair activities and tumor-associated pathways.^55^ For example, some USPs can directly participate in the activation of the NFκB pathway and thus positively regulate tumorigenesis,^58^ and some can indirectly inhibit NFκB activation and thus promote the invasive and migratory activity of BC cells.^59^ Some USPs can directly or indirectly inhibit or stabilize p53,^60^ and some USPs play a role in regulation of the stability of c-Myc during tumorigenesis.^61^^,^^62^

**Figure 3:**
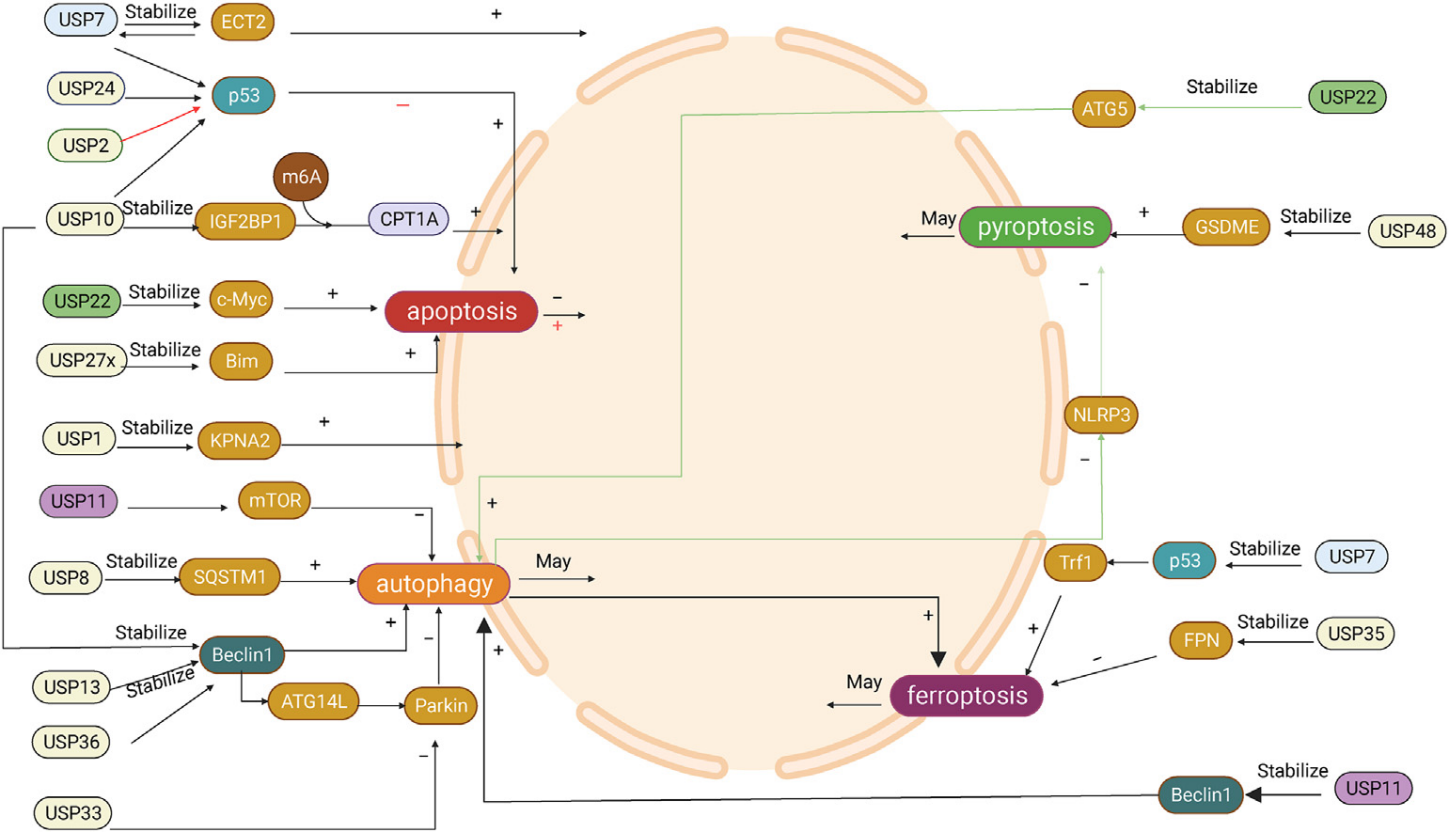
The role of USPs in PCD in BC. This figure summarizes the mechanisms of USPs in PCD-related proteins or pathways in BC, which were discussed in this review article (created with BioRender.com). (Note: USP: ubiquitin specific protease; ATG14L: autophagy-related gene 14-like protein; NLRP3: innate immune receptor protein (NOD-, LRR- and pyrin domain-containing 3); GSDME: gasdermin E; IGF2BP1: insulin Like growth factor 2 mRNA binding protein 1; ECT2: epithelial cell transforming factor2; CPT1A: carnitine Palmitoyltransferase 1A; SQSTM1: sequestosome 1; m6A: N6-Methyladenosine; mTOR: mechanistic target of rapamycin; KPNA2: karyopherin alpha2; ATG5: autophagy-related gene 5; Trf1: transferrin receptor 1).

Recently, the number of studies on USPs in BC has gradually increased. For example, USP7 promotes BC by stabilizing the epithelial cell transforming factor2 (ECT2) through deubiquitination^63^; USP1 promotes BC metastasis^64^; USP10 binds and deubiquitinates IGF2BP1, stabilizes it and then modifies CPT1A in an m6A dependent manner to modify CPT1A, thereby promoting BC metastasis.^65^ The mechanisms by which USPs are associated with PCD in tumor cell development were also elucidated, e.g., inhibition of USP1 induces apoptosis and autophagy in hepatocellular carcinoma^66^; depletion of USP35 increases sensitivity to cisplatin-induced apoptosis in NSCLC^67^; and USP35 can stabilize the RRS in NSCLC cells by RRBP1 to mitigate endoplasmic reticulum (ER) stress-induced apoptosis.^68^ Next, we will briefly describe the structure and function of USPs and describe the possible mechanisms of USPs in the PCD of different BC cells (Fig. 3).

### USPs and apoptosis in BC

Apoptosis, the most common form of PCD, is a process of controlled cell death that ultimately results from the cessation of cell growth and division. This process can be seen under the light microscope throughout the organelles, which are encapsulated in the intact plasma membrane, forming apoptotic vesicles that do not cause localized inflammation to develop.^69^ Biochemically, apoptosis is characterized by the involvement of caspases.^70^ Too little apoptosis leads to uncontrolled cell growth and division in cancer. Some ubiquitin-specific proteases deubiquitinate and stabilize apoptosis-associated pathway proteins.^71^

Aberrant expression of the proto-oncogene c-Myc may promote or inhibit apoptosis,^72^ and it has been shown that USP22 can increase c-Myc stability in cancer cells by deubiquitination and blocking proteasomal degradation.^61^ In contrast, c-Myc promotes apoptosis and accelerates cell turnover during cellular carcinogenesis, thereby promoting the progression of cells toward increasingly malignant phenotypes.^73^ Therefore, it is hypothesized that USP22 promotes cancer progression in BC cells by stabilizing c-Myc to promote apoptosis. USP22 was previously shown to play a regulatory role along with USP27x,^74^ which acts as a pro-apoptotic agent by stabilizing Bim proteins in NSCLC and melanoma,^75^ but its association with apoptosis has not yet been singled out. However, it deubiquitinated the cell cycle protein D1, thereby inhibiting cell growth in several HER2 treatment-resistant breast cancer cell lines.^76^

p53 is the first discovered tumor suppressor gene and plays a vital role in apoptosis induction. Because p53 is inactivated by gene mutations in most tumors, its typical role in causing apoptosis will not be able to function properly,^77^ and the tumors will develop further. Previous studies have shown that USP7, USP10, and USP24 can stabilize p53 expression and inhibit cancer formation.^78^, ^79^, ^80^ However, some studies have shown that USP2 stabilizes the expression of p53 through deubiquitination, and that there is a potential role for the p53 signaling pathway to participate in the inhibition of apoptosis and to promote the growth of breast cancer cells.^81^ This is not consistent with the earlier theory; the specific mechanism has not yet been clarified and needs further exploration.

### USPs and autophagy in BC

Autophagy is the process by which cellular components, such as organelles and macroproteins, are sequestered into lysosome-forming autophagic lysosomes for degradation. This fights diseases through self-digestion, protects cells, and plays a role in cell death.^82^ Autophagy can be both promoted and inhibited in tumors, which appears to depend on whether cancer cells have access to sufficient extracellular metabolites and energy^83^; when access is limited, autophagy promotes tumor growth. In eukaryotic cells, the UPS and autophagy are the two central protein hydrolysis systems, which are not completely independent of each other, and the relationship between them is gradually being revealed.^84^ Several DUBs have been reported to regulate autophagy by deubiquitinating the components of the autophagy pathway.

P62/SQSTM1 was identified as a signaling hub and selective autophagy receptor,^85^^,^^86^ and it is a multifunctional protein.^87^ p62 can serve as a storage site for ubiquitin proteins through preferential binding of polyubiquitin chains to form a new cytoplasmic structure, the “sequestosome”.^88^ Sequestosome 1 (SQSTM1) has multiple structural domains: Phox1 and Bem1p domain (PB1), UBA, and the LC3 interaction region.^89^ These domains mediate interactions with different signaling proteins and regulate various cellular functions.^86^^,^^90^ SQSTM1 is highly expressed in BC and plays an essential role in BC development,^91^ and USP8 can regulate SQSTM1 degradation and autophagy by deubiquitylating SQSTM1 at the K420,^92^ which has been shown to play a vital role in autophagosomal formation and autophagic flux.^93^ USP8 also regulates the SQSTM1 pathway and plays a role in BC development. This pathway plays an important role in BC. However, further investigation is required to elucidate the underlying mechanisms.

*Beclin1* is a gene that positively regulates autophagy and plays a role in autophagy induction.^94^ *Beclin1* is a tumor-suppressor gene, which is found at higher levels in normal breast cells than those in BC cells.^95^ USP10 and USP13 can mediate the deubiquitination of the autophagy-related protein Beclin1, stabilizing Beclin1 levels and exerting a tumor-suppressive effect. An increase in Beclin1 expression leads to an increase in the levels of the proto-oncogene p53, which inhibits tumor development.^96^ USP10 and USP13 may act as a suppressor in BC by promoting autophagy in tumor cells. USP36 regulates Parkin-dependent mitophagy partly through the Beclin1-ATG14L pathway.^97^ With Beclin1 as a key protein for autophagy,^98^ we can speculate that USP36 and USP33 may also regulate autophagy role in BC. USP33 can directly target parkin RBR E3 ubiquitin protein ligase (PRKN), and knockdown of USP33 enhances PRKN-mediated mitophagy,^99^ providing a new therapeutic strategy for the treatment of Parkinson's disease. USP33 may play an important role in the treatment of BC. Therefore, further studies are warranted in this regard.

Multiple studies suggest that mTOR may be a central regulator of autophagy.^100^^,^^101^ Moreover, some studies have shown that USP11 can inhibit cell autophagy through the ERK/mTOR pathway, which promotes the proliferation and metastasis of cancer cells.^102^ The ERK signaling pathway also plays a crucial role in BC. Additionally, ERK1/2 can inhibit the phosphorylation of USP11 and thus downregulate the level of cytoplasmic p21, which can play an oncogenic and pro-cancer role in BC.^103^ However, whether USP11 can inhibit autophagy through the ERK/mTOR pathway and play a key role in BC development needs further exploration.

### USPs and ferroptosis in BC

Theoretically, because ferroptosis is a reactive oxygen species-dependent form of cell death, and malignant cells have a higher ROS load, it is reasonable to hypothesize that cancer cells may have a higher propensity for ferroptosis. A growing body of research suggests that ferroptosis may be an adaptive response that removes damaged cells from the environment, acting as a tumor suppressor in the tumor environment.^104^^,^^105^ A variety of tumor suppressors and signaling pathways play a role in regulating ferroptosis,^106^ and USPs, which use these factors and pathway proteins as substrates, may also regulate ferroptosis.

USP7 forms a new pathway with p53/Tfr1 in rat cardiac cells after ischemia/reperfusion and activates this pathway, deubiquitinates and stabilizes p53, and promotes ferroptosis.^107^ USP35 regulates ferroptosis in lung cancer by targeting FPN,^108^ a key transferrin in mammals. Moreover, it has been shown that the expression of USP11 is increased in neuronal cellular ferroptosis, and USP11 regulates autophagy-dependent ferroptosis after spinal cord ischemia/reperfusion injury through the deubiquitination of Beclin1,^109^ which limits recovery from this disease. Moreover, the induction of ferroptosis in BC is beneficial in BC treatment.^34^ Whether USP11 plays a role in the treatment of breast cancer through this pathway, and whether there are other USPs that are critical for ferroptosis in breast cancer cells remains to be further investigated.

### USPs and pyroptosis in BC

Pyroptosis is a form of PCD associated with the inflammatory response, and its biochemical features are mainly marked by the formation of inflammatory vesicles.^110^ Unlike apoptosis, pyroptosis occurs more rapidly and violently and is accompanied by the release of multiple pro-inflammatory factors.^111^ The focal death-induced plasma membrane rupture-released molecular-damage-associated molecular patterns (DAMPs)^112^ contribute to the tumorigenic potential of inflammatory vesicle activation on the one hand,^113^ and on the other hand limit tumor cell survival and thus slow cancer progression.^114^ These contradictory actions have not been fully explored in BC.^115^ The innate immune receptor protein NLRP3, together with the adapter protein ASC and caspase-1, form the NLRP3 inflammasome,^116^ which mediates the production of a number of cytokines and plays a role in the pyroptosis of BC cells.^117^ The activation of the NLRP3 inflammasome was found to be regulated by deubiquitinating proteases, but by factors upstream of the inflammasome rather than by the inflammasome itself.^118^

The central mediators of pyroptosis are proteins from the gasdermin family.^119^ USP48 binds to gasdermin E (GSDME) and deubiquitinates the K48 junction at the K120 and K189 sites to stabilize GSDME, which sensitizes cancer cells to focal death and improves the response to immunotherapy.^120^ USP22 inhibits the activity of the NLRP3 inflammatory vesicle by promoting ATG5-mediated autophagy, which is the main mechanism to reduce the ubiquitination of the K27 and K48 site junctions and thus stabilize ATG5.^121^ Few studies investigated the relationship between USP and pyroptosis and the relationship between pyroptosis and BC. An in-depth study of the relationship among these three factors will expand our understanding of BC treatment and provide innovations in its prevention and treatment.

### USPs and other PCD pathways in BC

Necroptosis is a form of PCD that results in cell death via intracellular signaling-regulated RIPK1-driven formation of complex IIB.^7^ RIPK1,^7^ Z-DNA-binding protein 1 (ZBP1),^122^ and Fas-associated protein (FADD)^123^ are key components of necroptosis in BC cells. The exogenous E3 ubiquitin ligase, MKRN1, mediates FADD ubiquitination to protect against cellular overkill.^123^ In contrast, in BC, it may play an opposing role; however, no studies have shown that a specific USP acting on the above targets affects necroptosis.

Anoikis is a PCD mechanism that occurs when cells detach from the correct extracellular matrix, which prevents the shedding of epithelial cells from colonizing in the incorrect place, leading to disease.^124^^,^^125^ Many related proteins and transcription factors in BC enhance the sensitivity and resistance to anoikis.^126^ Resistance to anoikis is more pronounced in triple-negative breast cancer,^124^ thus causing metastasis of cancer cells and leading to a poor prognosis. Whether it is possible to affect the stability of some proteins through the ubiquitin-proteasome system and thus regulate anoikis to achieve a therapeutic effect remains to be determined in further studies.

This study examined the effects of USPs on apoptosis and autophagy in BC, two of the most prevalent PCDs. Moreover, this study explored the relationship between USPs and PCDs, which contribute to the development of BC and treatment resistance. However, this study did not address the role of USPs in necroptosis or anoikis. Furthermore, the USP and PCD processes may result in different BC outcomes, depending on the “double-edged sword” effect of the PCD or the target of the USP. Thus, it has been established that the relationship between USP and PCD can influence the development of BC. Therefore, effective therapies that induce or inhibit PCD and affect BC should be targeted (Table 2).

**Table 2:** PCD and USPs in BC.

Type of PCD	Type of USPs	targets	Role in PCD	Reference
Apoptosis	USP22	c-Myc	Promote	61,73
	USP27x	c-Myc、Bim	Promote	74,75
	USP7	P53	Promote	78, 79, 80
	USP10			
	USP24			
	USP2	P53	Inhibit	81
Autophagy	USP8	P62/SQSTM1	Promote	92
	USP13	Beclin1	Promote	96
	USP10			
	USP36	Beclin1, parkin	Inhibit	97
	USP33			99
	USP11	mTOR	Inhibit	102
Ferroptosis	USP7	p53/Tfr1	Promote	107
	USP11	Beclin1	Promote	109
	USP35	FPN	Inhibit	108
Pyroptosis	USP48	GSDME	Promote	120
	USP22	ATG5	Promote	121

(Note: ATG5: autophagy-related gene 5; BC: breast cancer; FPN: ferroportin; GSDME: gasdermin E; mTOR: mechanistic target of rapamycin; PCD: programmed cell death; SQSTM1: sequestosome 1; Trf1: transferrin receptor 1; USPs: ubiquitin specific proteases).

## Conclusions and future perspectives

PCD is fundamental to the maintenance of cellular redox homeostasis, normal tissue development, and human health. PCD dysregulation is a substantial cause of BC development. Because the levels of PCD-promoting and PCD-resistant proteins can determine the life and death of a cell, the regulation of protein turnover is of particular importance. Several studies have shown that the ubiquitin-proteasome system plays an important role in protein turnover, linking the ubiquitin proteasome and programmed cell death.

BC has become one of the most common cancers worldwide and is resistant to therapy. Thus, the search for new therapeutic targets has become a major goal. The induction or inhibition of PCD events by deubiquitination-associated proteins via USPs may be a very effective therapeutic approach for BC. USP10, USP11, and USP22 have been shown to regulate tumor growth and metastasis by affecting PCD in some cancers for therapeutic purposes. USP7 and USP11 affect several types of PCD, such as pyroptosis and ferroptosis. This depicts that USP has more than one substrate and can crosstalk with multiple PCD pathways; for example, autophagy-dependent ferroptosis and autophagy-dependent pyroptosis. Inhibition of USP1 induces both an increase in endogenous apoptosis and autophagy, triggers protective autophagy, and decreases apoptosis by causing an increase in AMPK phosphorylation,^66^ and the results may be either mutually reinforcing or antagonistic. The p53-related pathway regulates apoptosis, autophagy, and ferroptosis. Its role extends beyond the typical regulation of apoptosis. Mechanistic studies of the USP pathway, which regulates a wide range of PCD pathways, can be performed in anoikis and necroptosis, leading to potential new advances.

Currently, the role of USP in the regulation of PCD in BC is poorly understood, and the development of drug resistance in BC remains a major obstacle to the effectiveness of cancer treatment. The discovery of new targets and investigation of compounds that can specifically target proteins that regulate PCD could substantially improve the efficiency of BC treatment.

## CRediT authorship contribution statement

WY and SX collected relevant literature and received and drafted the manuscript. XZ and JF reviewed the manuscript and revised it. All authors contributed to the manuscript and approved its submission.

## Conflict of interests

No potential conflicts of interest were disclosed.
